# Mechanical, microstructural and fracture studies on inconel 825–SS316L functionally graded wall fabricated by wire arc additive manufacturing

**DOI:** 10.1038/s41598-023-32124-3

**Published:** 2023-03-31

**Authors:** T. S. Senthil, S. Ramesh Babu, M. Puviyarasan

**Affiliations:** 1 Department of Mechanical Engineering, Panimalar Engineering College, Anna University, Chennai, Tamil Nadu India; 2 Department of Mechanical Engineering, Sri Venkateswara College of Engineering, Sriperumbudur, Tamil Nadu India

**Keywords:** Mechanical engineering, Materials science

## Abstract

This paper presents a novel method that uses the cold metal transfer based wire arc additive manufacturing process to fabricate functionally graded Inconel 825–SS316L walls. The optical micrograph of Inconel 825 exhibits continuous and discontinuous dendritic structures. The SS316L region comprises 5% of δ-ferrite in primary austenitic (γ) dendrites which was confirmed by the Cr_eq_/Ni_eq_ ratio of 1.305. The functionally graded interface reveals a partially mixed zone with a transition from the elongated dendrites to fine equiaxed dendrites. The tensile properties of the fabricated wall were determined at room temperature using specimens extracted from Inconel 825, SS316L, and the interface regions. The morphology of the tensile tested specimens revealed significant plastic deformation, indicating ductile failure. The fracture toughness of the wall was experimentally investigated by employing the crack tip opening displacement (CTOD) test. The fracture morphology exhibited a ductile mode of fracture with striations perpendicular to the direction of crack development. Elemental mapping revealed that there was no evidence of elemental segregation on the fractured surfaces, and the elements were uniformly dispersed. The CTOD measures 0.853 mm, 0.873 mm on the Inconel 825 side and the SS316L side respectively. The test results confirm that both the Inconel 825 and SS316L sides have good fracture toughness.

## Introduction

Throughout history, the ability to comprehend and manipulate materials has been critical to the advancement of technology. Today’s scientists and engineers understand the value of novel materials in terms of the economy and the environment. Functionally graded materials (FGMs) are sophisticated and extremely functional zones in a part that exhibit a constant change in elemental composition, resulting in novel and customised mechanical or thermal properties^1^. The ability to develop materials with enhanced properties that are suitable for a variety of applications, including aerospace, marine, nuclear engineering, and high-temperature protective coatings has increased attention to FGMs significantly^2^. Size and structural characteristics are two factors that can be used to classify gradient materials. Gradients can be bulky or thin-section (like surface coatings), which require distinct processing techniques. They are separated into two groups: continuous and discontinuous, depending on the structure. In materials with discontinuous gradients, the microstructure or chemical composition varies gradually, and the interface is typically perceptible and observable. Contrarily, in materials with continuous gradients, the chemical composition or microstructure continuously alter with position, making it nearly impossible to perceive a distinct boundary as the interface across the graded structure^3^.

Recently, many researchers have focused on metal-based FGMs. Sobczak et al.^4^ discussed the fundamental manufacturing processes for metal-based FGMs. Domack et al.^5^ used three distinct fabrication techniques to create Inconel 718-Ti–6Al–4V FGM. It was reported that the samples of laser direct metal deposition showed notable elemental segregation and coarse dendritic microstructures. Using cold metal transfer welding Tian et al.^6^, examined the mechanical and microstructural behaviour of dissimilar Ti–6Al–4 V and AlSi5 alloys and found a crack in the interface layer. It originated at the interface layer and extended to the Al side as a result of the difference in alloy shrinkage between Al and Ti. Niendorf et al.^7 ^ reported that selective laser melting (SLM), is used to make stainless steel parts with a variety of local functionalities. They found that a steep microstructural gradient leads to distinct local mechanical properties. It has been demonstrated that directed energy deposition could be used to fabricate FGMs from Inconel 625 and SS304L and that the characteristics and thermodynamic models of these materials have been investigated by Carroll et al.^8^. Inconel alloys are difficult to work with because they tend to harden during processing and adhere to cutting tools^9^^,^^10^. Inconel825 and SS316L were austenitic materials with high chromium content, which provides excellent resistance to high-temperature corrosion^11^. Solidification cracking can occur during the fusion welding of these two materials. The cold metal transfer (CMT)–based WAAM process can be used to avoid this problem^12^. The CMT process is a modified gas metal arc welding process that was developed in 2004 by Fronius International, Austria. As the name implies, CMT based WAAM is a process in which molten metal is transferred with a very small heat input to fabricate the wall. The smart automation system and a weld head with an embedded controller pull the filler away from the melt pool when it makes contact, mechanically transferring the molten metal, thereby reducing the amount of heat involved. Moreover, to increase the cooling rate, fins made of aluminium and blowers are installed underneath the substrate holder. This enhances the quality of printed parts. Additionally, the CMT based WAAM process provides an unwavering arc, improved process steadiness, and limited dilution^13^. Therefore, CMT based WAAM is a highly specialised additive manufacturing process with enormous potential for mass production due to its higher deposition rate, which enables faster fabrication than any other additive manufacturing process.

Fracture toughness is an important property that indicates how resistant it is to cracks and estimates the amount of stress needed to propagate a flaw that already exists. During processing, manufacturing, or servicing a component, flaws cannot be completely avoided.

It is believed that the crack tip opening displacement (CTOD) is the most important criterion for assessing the fracture toughness of steel weldments. Leng et al.^14^ explored the correlation between fracture toughness and morphology of S335G10 + N weldments using CTOD. It has been found that the CTOD reduces as the average grain size increases. Guo et al.^15^ experimented with different regions of 9Cr and Cr–Mo–V weldments. They observed that the fracture toughness of the Cr–Mo–V side was significantly greater than the 9Cr side. Wang et al.^16^ investigated the rupture characteristics and morphology of weldments made of A508 and 316L stainless steel. Ductile fracture involving micro-pore nucleation, growth, and coalescence was reported. Additionally, it was found that the weldments fracture path was significantly influenced by the orientation of columnar austenite crystals in the weldment. Li et al.^17^ investigated the fracture characteristics of Fe_3_Al and Cr18-Ni8 weldments. It was found that the crack opening is located in the Fe_3_Al side, which contains a significant number of deformations. Only a small number of cracks have spread horizontally to the fusion zone and terminated at the weld. The majority of the cracks have continued to extend alongside the fusion zone. The cracking in a functionally graded coating was investigated by Bao et al.^18^. The impact of material non-homogeneities on stress intensity factors was explored in their studies. The CTOD analysis was used to assess the fracture toughness of the material at room temperature using the BS7448 standard, despite several other methods. The J-Integral method employing the line integral is challenging and unreliable. The CTOD method derived from the crack mouth opening displacement is more suitable for calculating fracture toughness^19–24^.

Though the CMT based WAAM is capable of fabricating defect-free components, flaws may occur during its service in industries like oil and gas transportation. For this reason, assessing the fracture behaviour of components is crucial to ensure their safety. The fracture toughness of FGM walls fabricated with CMT based WAAM has not been reported. This research aims to assess the fracture behavior of Inconel 825-SS316L functionally graded walls fabricated using CMT based WAAM. The fabricated walls were analysed for microstructure, fracture morphology, and inclusions near the fracture zone to ascertain their fracture toughness.

## Materials and methods

Inconel 825 has a stable austenite structure and contains small amounts of molybdenum, titanium, and copper. The elemental composition of the alloy is designed to perform in extremely corrosive environments. The high nickel content provides ample resistance to stress-corrosion cracking. Nickel, together with molybdenum and copper, acts as a protective barrier by reducing the presence of harmful acids in the environment. Chromium provides resistance to corrosion and undesirable oxidising agents. The titanium stabilises the alloy against sensitization, allowing it to withstand intergranular deterioration. At cryogenic temperatures, the austenitic nature of the SS316L prevents sensitization [23, 24]. The chemical compositions of wire electrodes used in the fabrication process were ascertained by spectroscopy and are summarised in Table 1.

**Table 1:** Chemical Composition of Inconel 825 and SS316Lwire electrode.

Electrode material	Inconel 825 (wt%)	SS316L (wt%)
Ni	45.25	13.4
Cr	23.5	17.5
Cu	3.35	–
Mo	3.3	2.6
Ti	0.9	–
Mn	0.95	1.9
Si	0.5	0.7
Al	0.2	–
C	0.04	0.02
S	0.01	0.025
P	–	0.03
Fe	22	Balance

**Figure 1:**
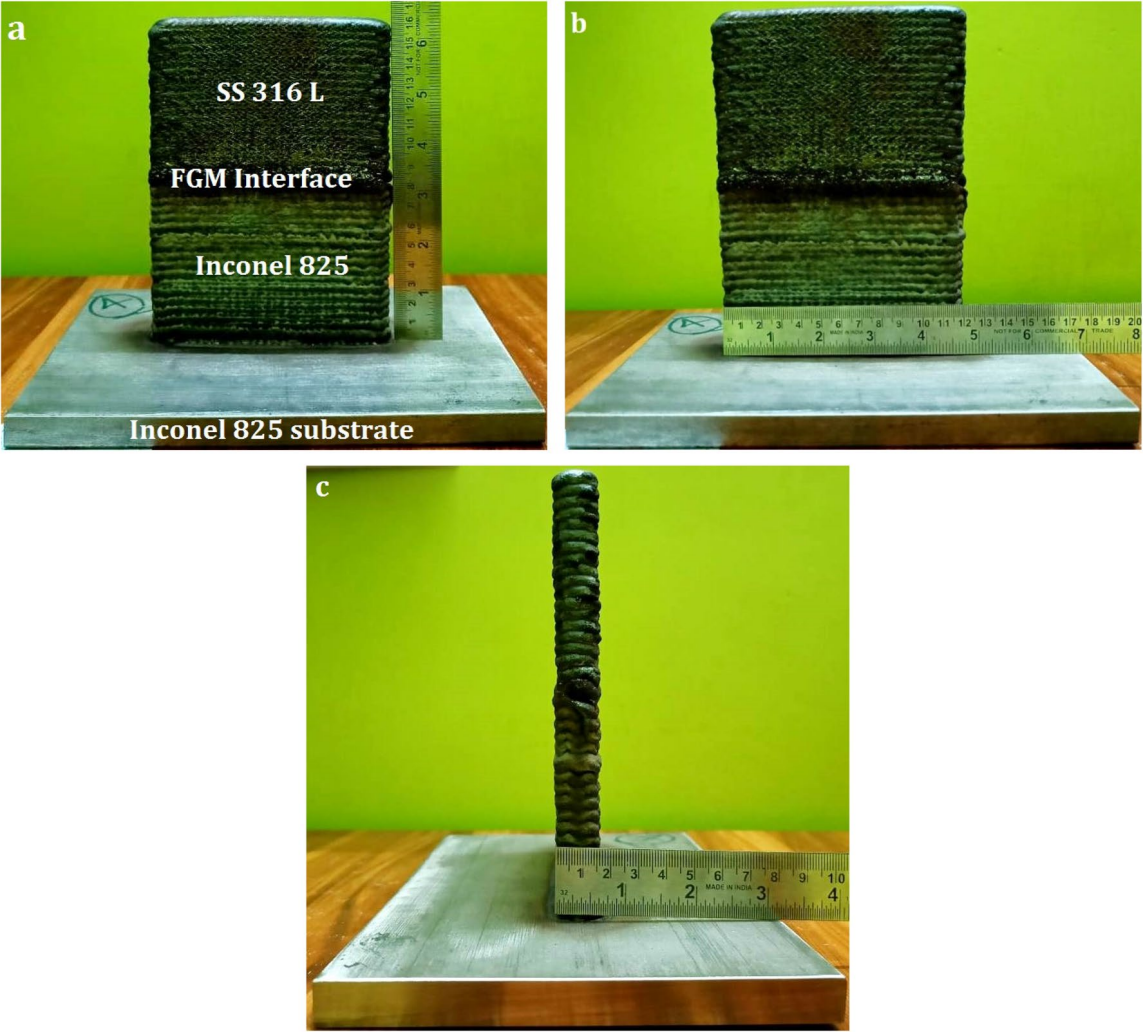
As-built wall showing (**a**) height (**b**) width (**c**) thickness.

The as-built wall in Fig. 1 measures 160 mm in length, 120 mm in width, and 16 mm in thickness. It was built by droplet transfer of twenty layers of Inconel 825 followed by twenty layers of SS316L. Droplet transfer was accomplished using 1.4 mm diameter filler wires. Each layer is built to a height of 4 mm. Based on our previous study^25^, the variables in the CMT-WAAM process have been selected (Table 2).

**Table 2:** CMT-WAAM process parameters.

Deposited material	Filler wire feed rate (mm/min)	Torch travel speed (mm/min)	Deposition current (A)	Flow rate of argon gas (l/min)	No. Of layers
Inconel 825	5000	150	146	13	20
SS316L	3800	150	113	13	20

The molten metal is transferred with a very small heat input to form the wall. To increase the cooling rate, aluminium fins as well as a fan are installed underneath the substrate mount. The wall construction procedure was completely programmed and carried out by a fully automated CMT robot that continuously constructed the wall without interruption. As a result, there is no downtime between the layers that are constructed. The process was paused after building 20 layers of Inconel 825 wall to switch the wire electrode from Inconel 825 to SS316L. Before depositing the SS316L material, the top layer of the Inconel 825 wall was heated with a gas welding torch until it was red hot to ensure strong adhesion at the interface.

**Figure 2:**
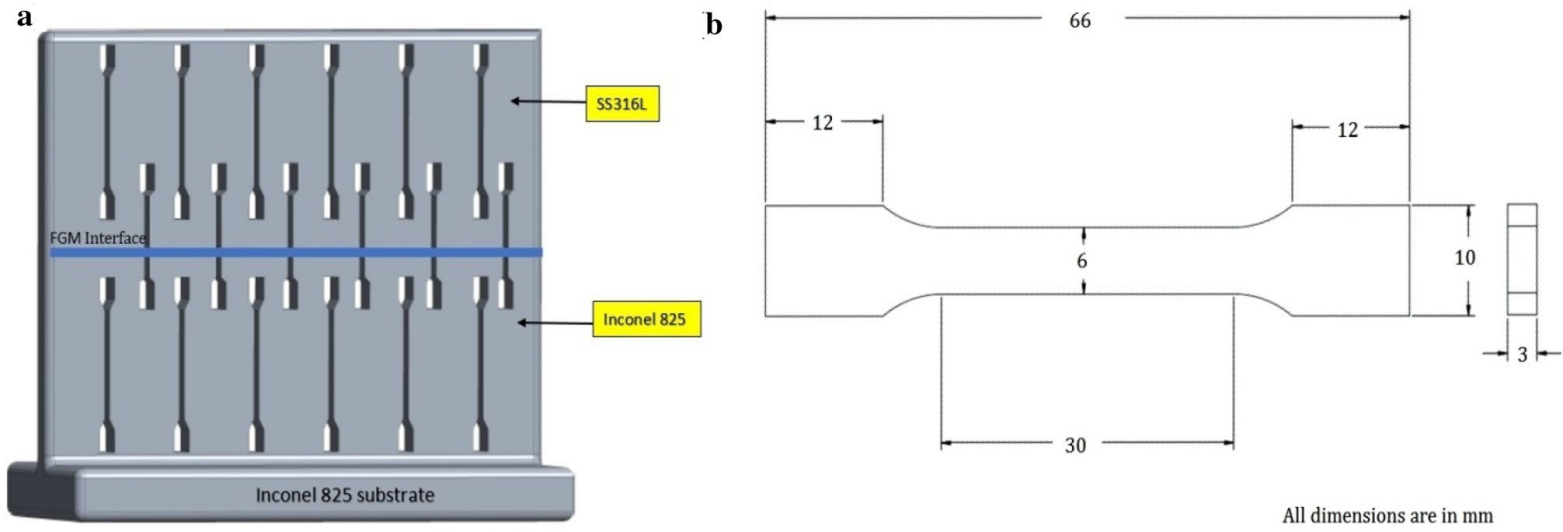
(**a**) Location of tensile specimens (**b**) Specification of tensile specimens.

The microstructure of the as-deposited wall was examined using optical microscopy. The microstructural evaluation was carried out in accordance with the ASTM E3-11 (2017) standard of the American Society for Testing and Materials. The extracted specimens were exposed to 10 ml of HCL and 3 ml of H_2_O_2_ for 15 s. In order to find the Ni/Cr equivalency ratio of SS316L specimens the EDS analysis was carried out. The room temperature tensile test was carried out according to the ASTM E8 standard on the specimens cut along the vertical direction (Fig. 2a) using the wire-cut electrical discharge machine (WEDM)^26–28^. The specification of the tensile specimen is shown in Fig. 2b.

**Figure 3:**
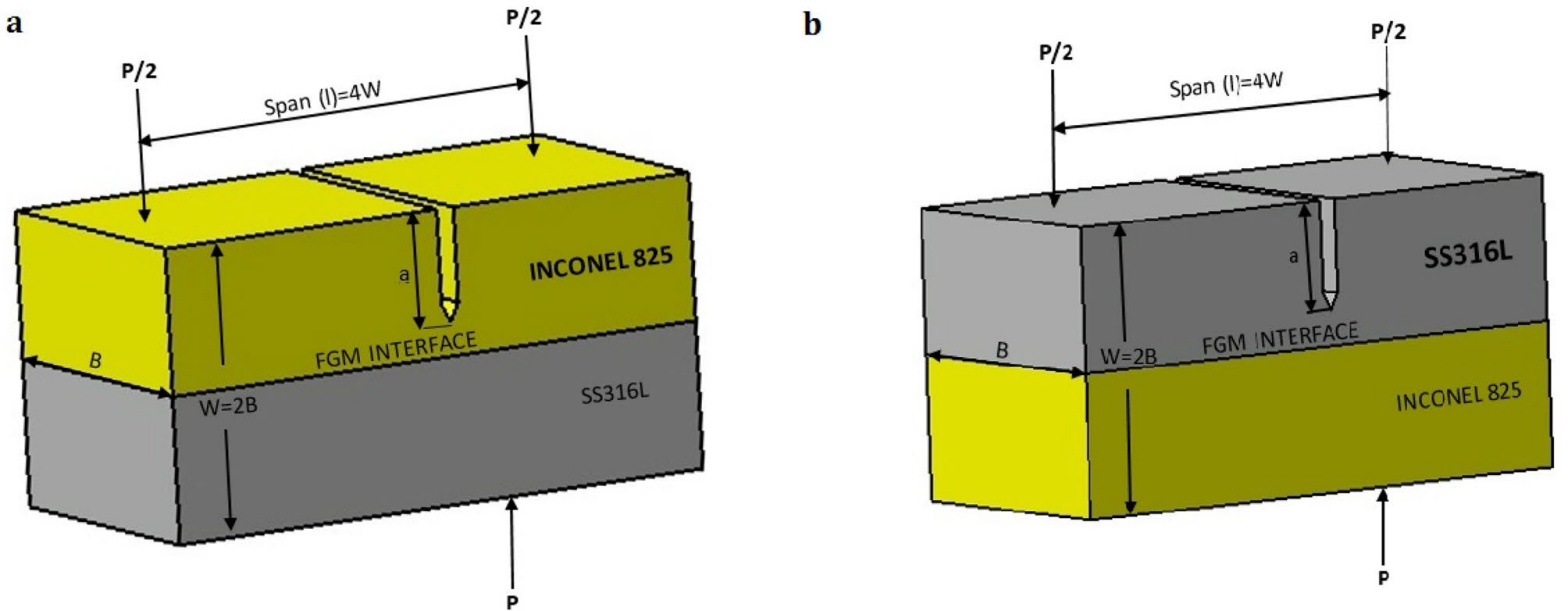
Fracture toughness specimen notched at (**a**) Inconel 825 (**b**) SS316L.

The evaluation of fracture toughness was carried out using the CTOD test, in accordance with ASTM E1290-89^29^. Two specimens were prepared by sectioning the fabricated walls along the interface, one with a notch on the Inconel 825 region (Fig. 3a) and the other with a notch on the SS316L region (Fig. 3b).

The L–T orientation extracts more energy than any other orientation, giving it greater fracture toughness. The notch is cut parallel to the specimen’s thickness direction. The notch’s machined depth was 45–55% of the specimen’s thickness, and the notch angle was 30°.To confirm that the test is independent of the notch root effect, a pre-cracking test was performed to produce a crack from the notch root. To nullify notch effects during the cracking process, the machined notch must be deep and narrow enough to have no notch root effect on the crack formation. In order to minimise damage to the surrounding region^30^ WEDM is recommended for machining the notch. According to the ASTM E-399 standard, the terminal pre-cracking force (P_f_) used to pre-crack the specimen is calculated using the Eq. (1)^25^.

1 $${\text{P}}_{{\text{f}}} = { }\frac{{0.5{\text{Bb}}^{2} {\upsigma }_{{\text{y}}} }}{{\text{S}}}$$

where S—loading span (mm), B—specimen thickness (mm), σ_y_—yield strength, b = W − a_o_, where W—specimen depth and a_o_—notch length.

The calculated terminal pre-cracking loads of 2.66 kN and 2.34 kN were applied to the Inconel 825 and SS316L specimens, respectively. Loading was accomplished at 2 mm/min. The pre-crack length on both the notched specimens measures 2 mm. After the specimen had been pre-cracked, the CTOD test was conducted. The displacement of the crack tip opening from its original position was determined using a crack-mouth clip gauge. The P-S curve was generated throughout the procedure (where P denotes applied load and S denotes crack tip opening displacement). Scanning electron microscopy (SEM) analysis was used to investigate the fractured surface of the specimens. Energy dispersive spectroscopy (EDS) elemental mapping investigates the elemental segregation at the fractured surfaces.EDS line scan analysis was performed to determine the presence of intermetallics or secondary phases at the fractured surface.

## Results and discussion

### The microstructure of the as-deposited wall

**Figure 4:**
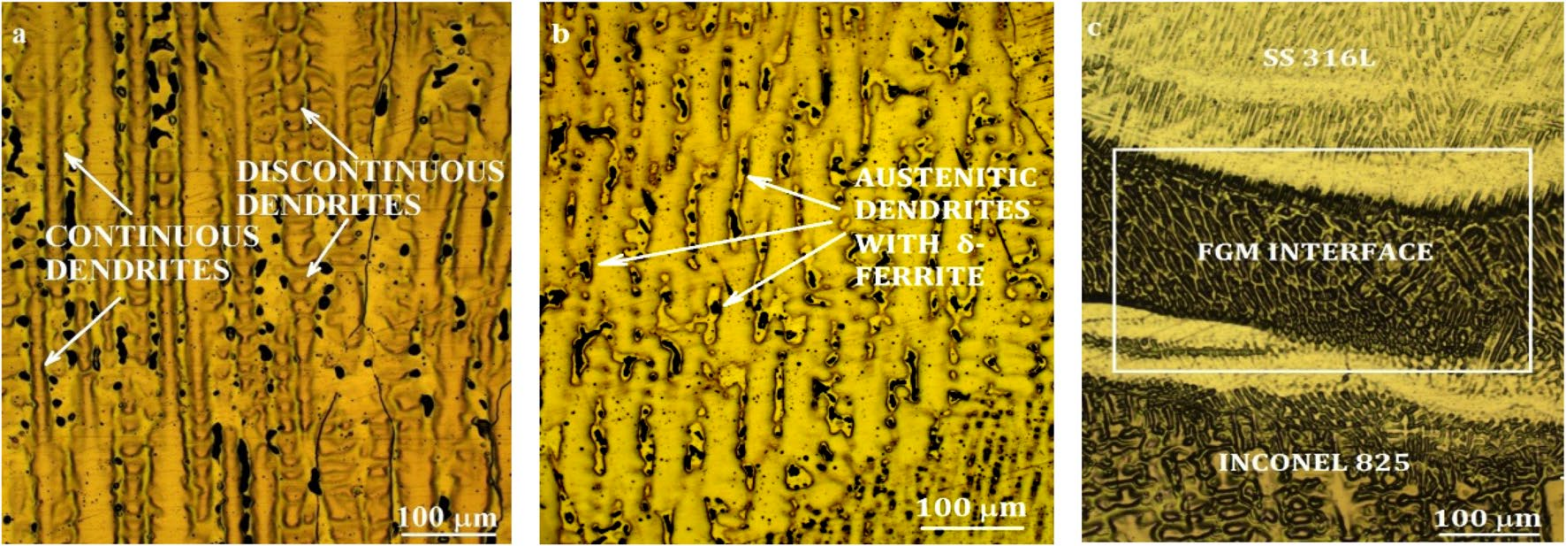
Optical micrograph of (**a**) Inconel 825 (**b**) SS316L (**c**) Interface.

The optical micrograph of Inconel 825 (Fig. 4a) depicts continuous and discontinuous cellular dendrites. Both microstructures exhibit the same growth direction and appear sequentially. At the grain boundaries, there were few secondary phases. Due to the alloy composition of Inconel 825, the development of carbides is unavoidable at high temperatures. Being a solid solution, it was predicted that many Ti (N, C) precipitates would occur in Inconel 825.As it is a cold metal transfer process, possibility of secondary phase formation is very less^31^. From the EDS analysis of SS316L, the Cr_eq_/Ni_eq_ was found to be 1.305. This confirms SS316L side comprises 5% of δ-ferrite in primary austenitic (γ) dendrites (Fig. 4b)^32,33^.

The micrograph of the functionally graded wall interface reveals a partially mixed zone (Fig. 4c). It also shows a microstructural transition from elongated dendrites to fine equiaxed dendrites. At the interface, none of the common flaws like cracks, partial fusion, or delamination are present.

### Tensile properties

**Figure 5:**
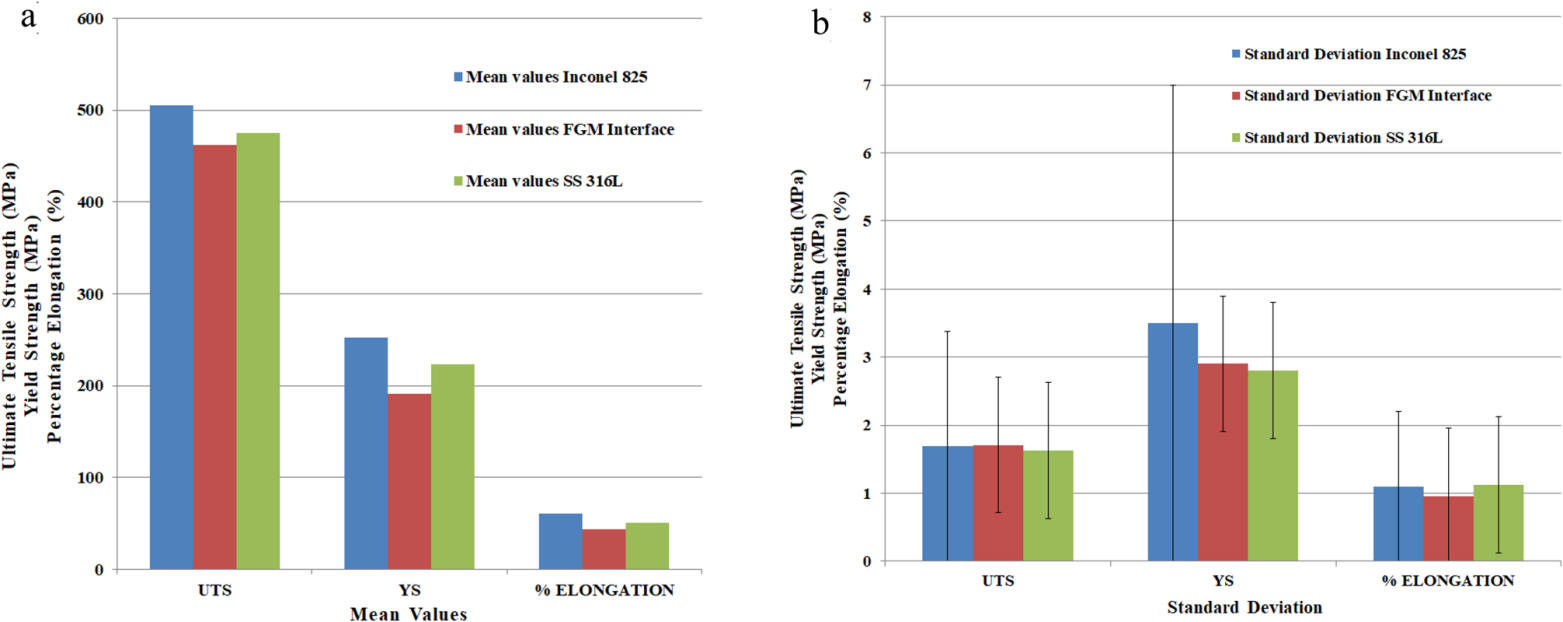
Tensile properties of as-deposited Inconel 825, Interface and SS316L (**a**) Mean values (**b**) Standard deviation with error bar.

The tensile properties of the fabricated walls were determined using the results of the tensile tests as shown in Fig. 5a,b. The tensile strengths of Inconel 825 and SS316L are comparable to those of wrought alloys^34^. The maximum values of standard deviation (UTS:0.35% and YS:1.51%) is much lower, which confirms that the results of the tensile test are within the acceptable limit.

The tensile properties (UTS, YS and percentage elongation) of the interface are slightly lower than Inconel 825 and SS316L. This may be due to the low heat input and faster cooling rates of the CMT-WAAM process that provides less time for elements such as Mo and Cr to diffuse, resulting in partial mixing^25^.

**Figure 6:**
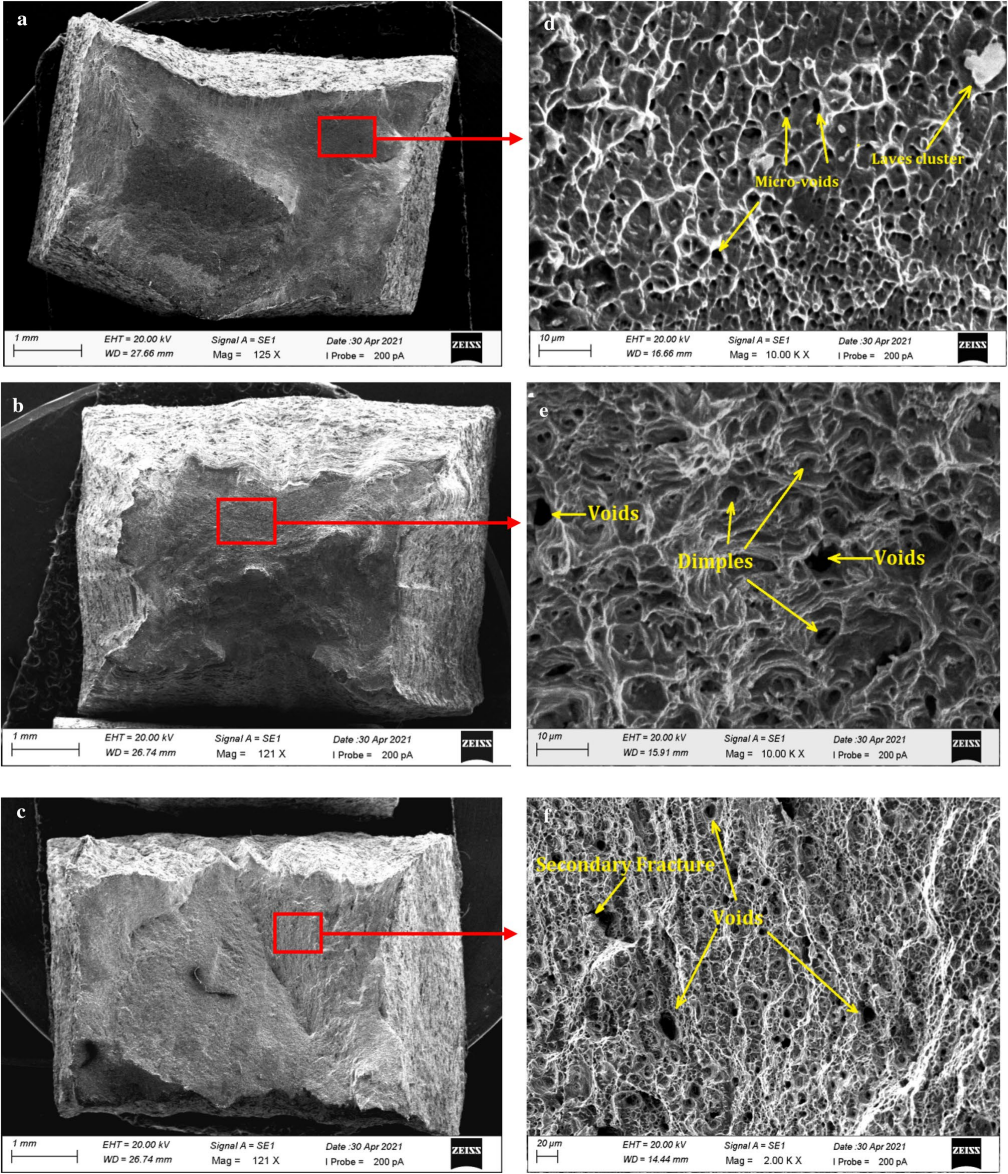
Fractured surface of (**a**) Inconel 825 (**b**) Interface (**c**) SS316L Magnified view of the necked region (**d**) Inconel 825 (**e**) Interface (**f**) SS316L.

Figure 6a–f shows the SEM micrographs of the fractured surface during the tensile test. Figure 6a–c reveals the necking area created by plastic deformation. The necked region of the Inconel 825, interface, and SS316L specimens are shown in higher magnification (Fig. 6d–f). In all the specimens large numbers of dimples were observed, indicating the failure was due to ductile mechanisms.

The tensile fractograph (Fig. 6d) of the Inconel 825 specimen indicates a fibrous ductile form of fracture. Scanning electron microscopy shows the presence of clustered laves phase and micro-voids despite the improved tensile characteristics. The fractograph of the interface (Fig. 6e) reveals that there was sufficient plastic deformation prior to failure, which indicates a ductile fracture. Fine dimples and small cavities were observed on the fractograph of the SS316L region (Fig. 6f) which confirms the ductile mode of fracture. Dimples are shallow openings unlike voids. Dimples produced by a micro-void coalescence process can be extremely shallow, with cups as small as several nanometres^35^.

### Evaluation of fracture toughness

A v-notch was cut in the L–T direction as the specimen absorbs more energy when the cracks grow in that direction. The experimental parameters and the measured CTOD values are listed in Table 3.

**Table 3:** Experimental parameters and CTOD value.

Sample geometry	SENB	SENB
Notch side	Inconel 825	SS316L
Orientation	L–T	L–T
Thickness (B) mm	10.24	10.20
Width (W) mm	20.02	20.06
Notch length (a_0_) mm	7.00	7.00
Terminal pre-cracking force (P_f_) kN	2.66	2.34
Number of cycles	39,684	36,802
Final crack length (a) mm	9.43	9.67
Maximum load value at fracture (P_max_) kN	5.37	4.55
Crack tip opening displacement (CTOD) mm	**0.853**	**0.873**
Fracture toughness (K_IC_)	**36 Mpa**$\sqrt {\mathbf{m}}$	**31.6 Mpa**$\sqrt {\mathbf{m}}$

Significant values are in bold.

**Figure 7:**
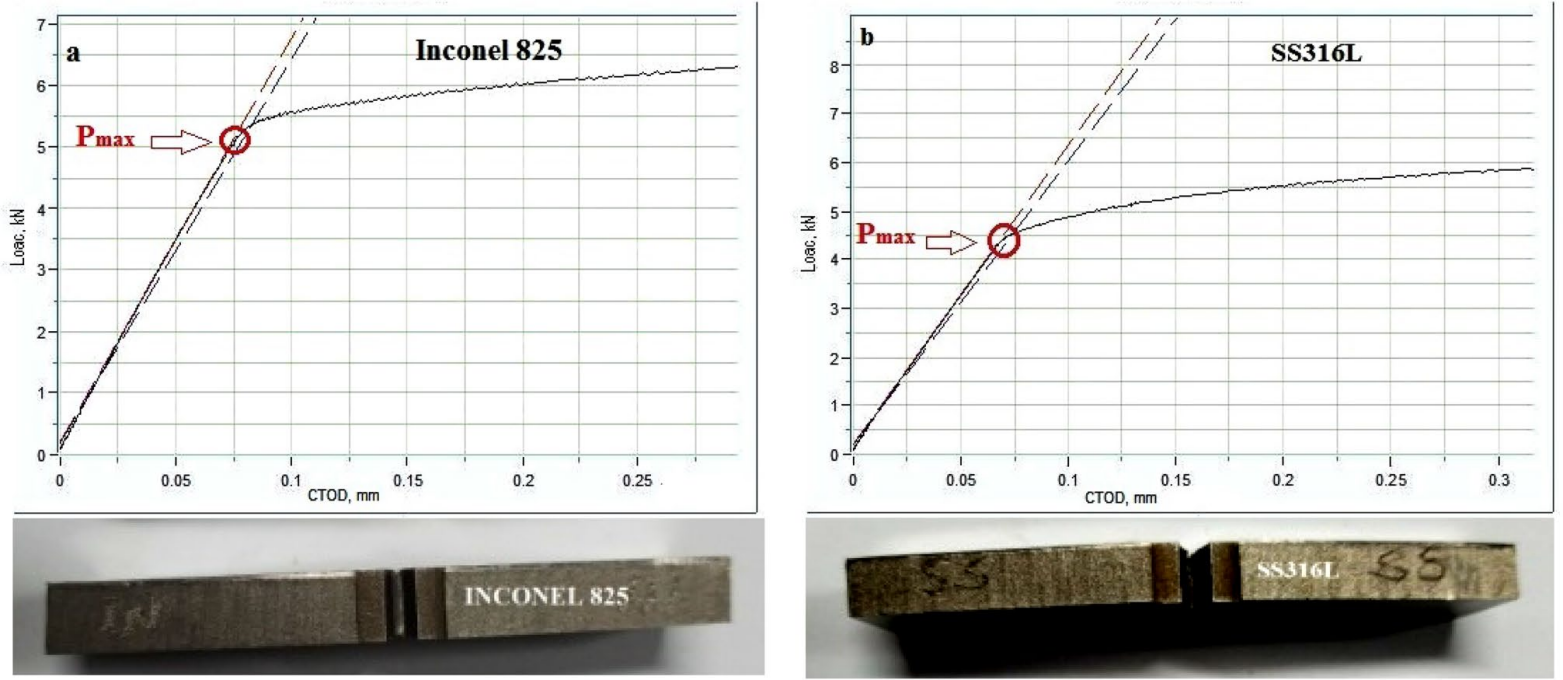
Crack tip opening displacement curve (**a**) Inconel 825 (**b**) SS316L.

The graphs in Fig. 7a,b show the P–S curves of the Inconel 825 side-notched specimen and the SS316L side-notched specimen, respectively. In both specimens, the maximum load value was reached, resulting in significant yielding and stable crack extension. The results in Table 3 show that the CTOD values differ only slightly between the two specimens. The maximum load value at fracture on the Inconel 825 side is 18% higher than the SS316L side. This is because of the higher concentration of nickel in Inconel 825 that improves the toughness and strength by refining the grain size^36^. The CTOD value on the SS316L side is 2.3% higher than the Inconel825 side indicating relatively faster crack propagation on the SS316L side. The fracture toughness values of walls are very similar to those of the cast versions of their base metals (Inconel 825 and SS316L)^37^^,^^38^.

**Figure 8:**
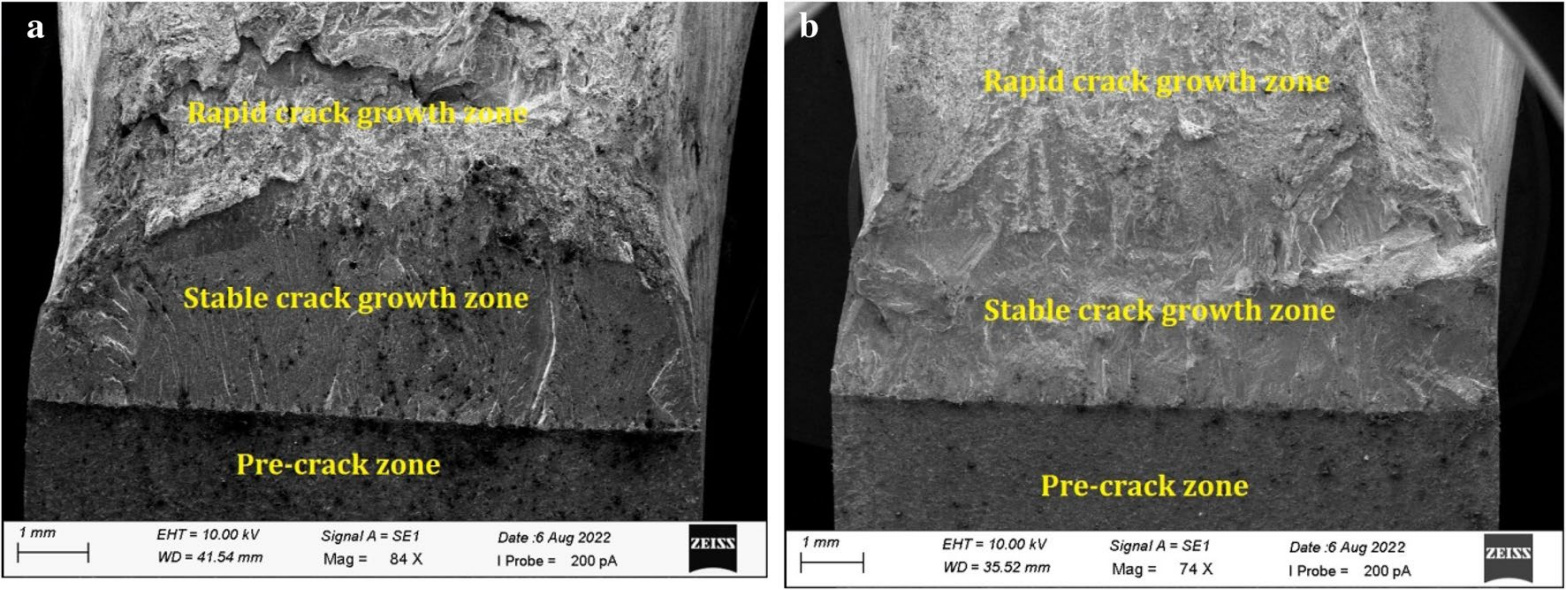
SEM images showing different zones of crack growth (**a**) Inconel 825 (**b**) SS316L.

The fracture surfaces of the single edge notched bend (SENB)specimens were investigated using SEM analysis. Figure 8a,b depicts the macro view of the fractured surface, which shows pre-crack, stable crack growth, and final fracture zones of Inconel 825 specimen and SS316L specimen respectively.

**Figure 9:**
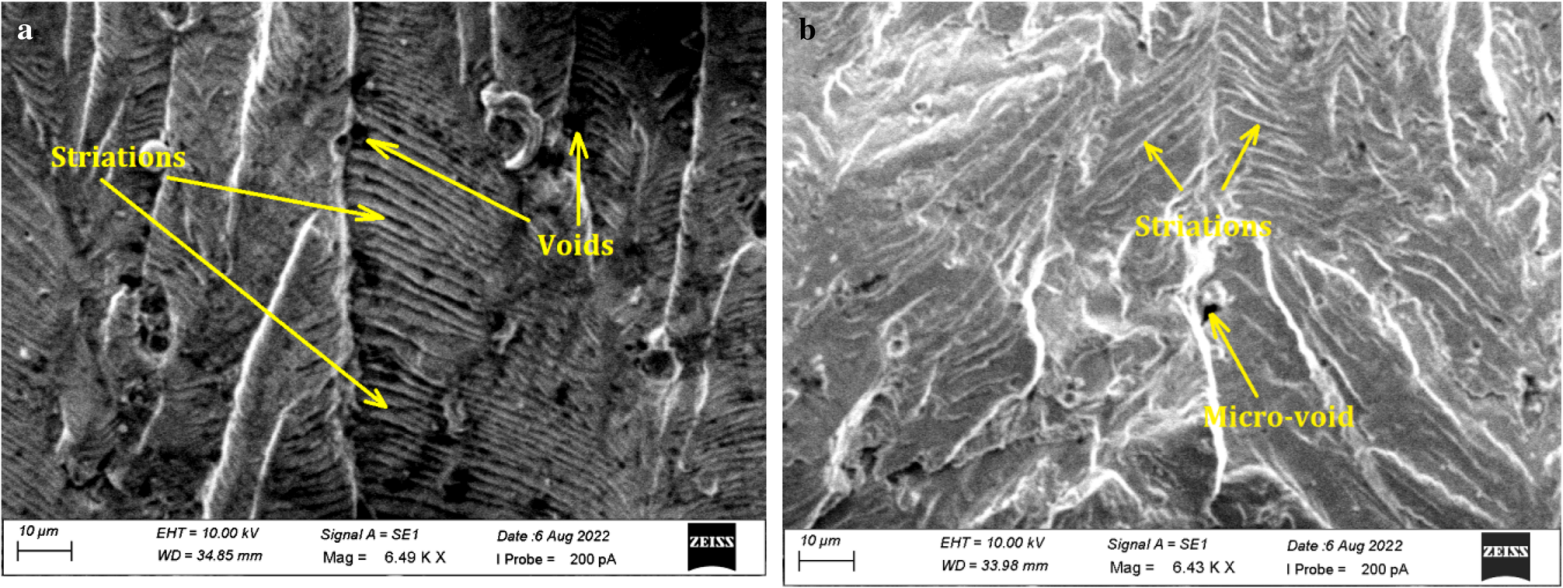
SEM micrograph showing striations (**a**) Inconel 825 (**b**) SS316L.

Figure 9a,b shows the striation on the fracture paths, which indicate the incremental growth of a crack and the direction in which the crack is propagating. Because of the complex state of loading in these materials, it is not possible to establish a direct relationship between striation spacing and crack growth in FGMs^39^.When the specimen is loaded to the level of producing voids, the local stresses at the crack-tip gain strength. The voids continue to expand and become connected to the primary crack.

**Figure 10:**
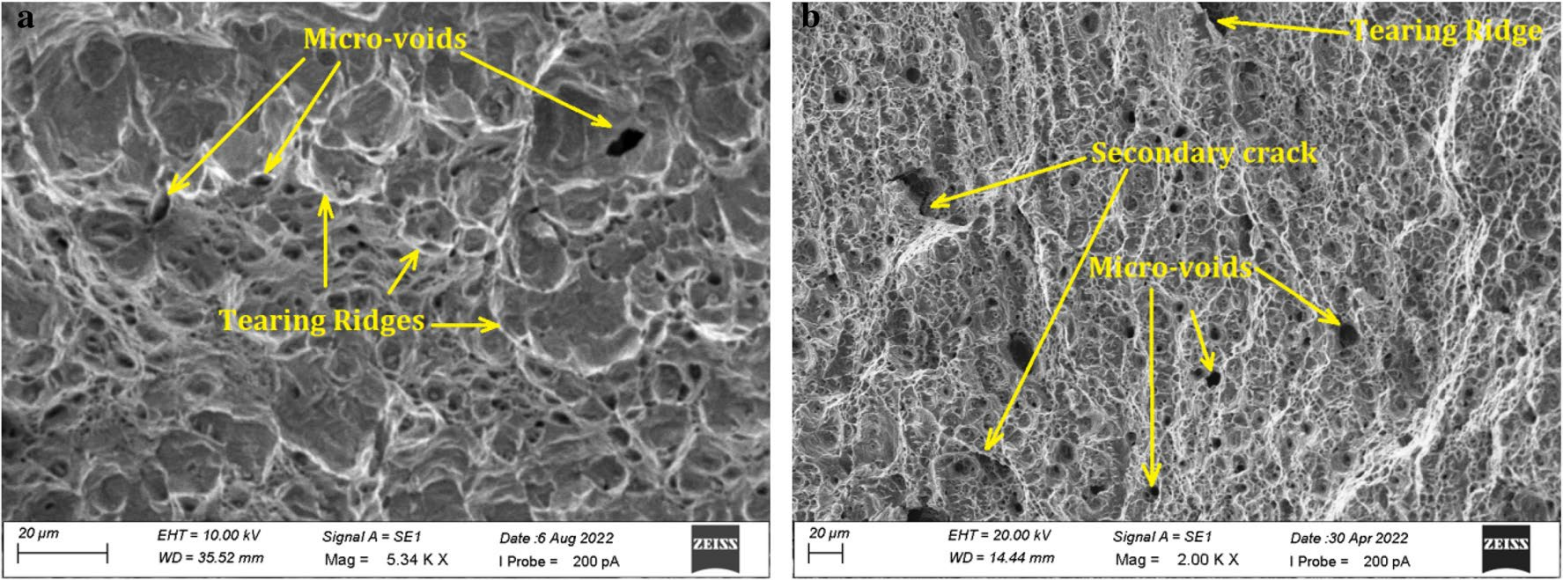
SEM micrograph of rapid fracture surface (**a**) Inconel 825 (**b**) SS316L.

Figure 10a,b depicts the ductile fracture morphology of the side notched specimens, which indicates that micro-void nucleation and formation occurred prior to the initiation of the crack opening process. The nucleation, growth, and coalescence of micro-voids can be used to characterise the mechanism of crack growth in ductile materials.

### Elemental mapping of the fractured surfaces

**Figure 11:**
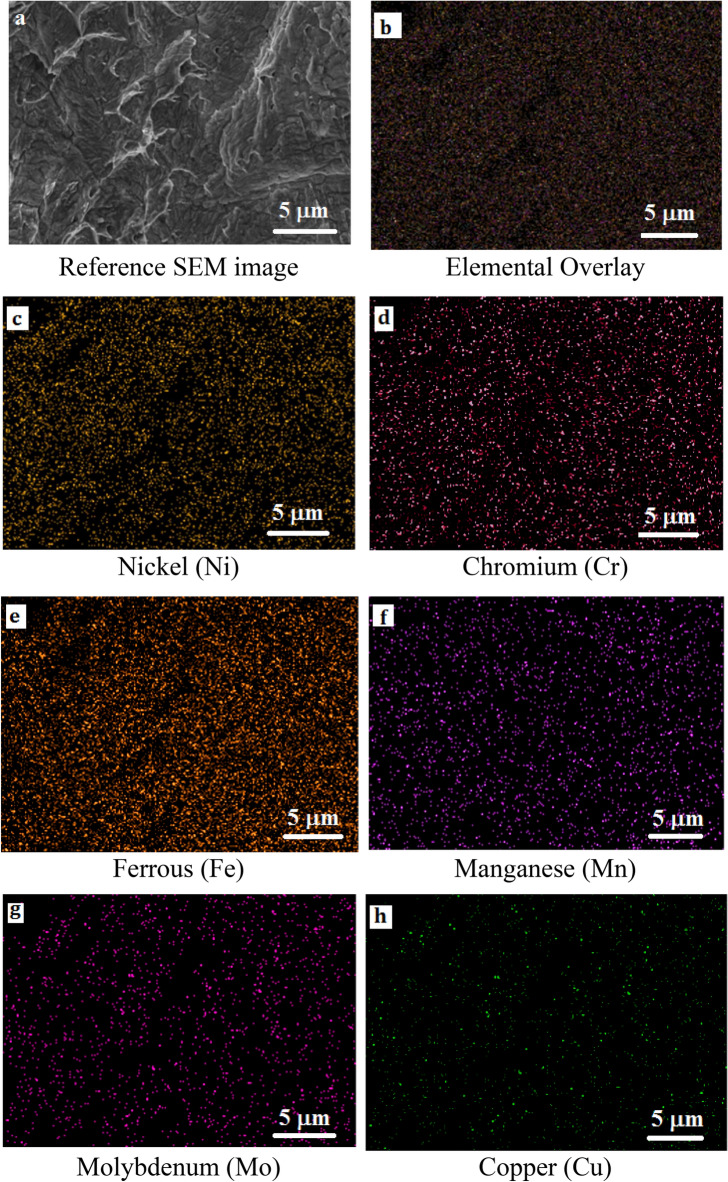
(**a**–**h**) EDS Elemental Mapping of the fractured Inconel 825 region.

**Figure 12:**
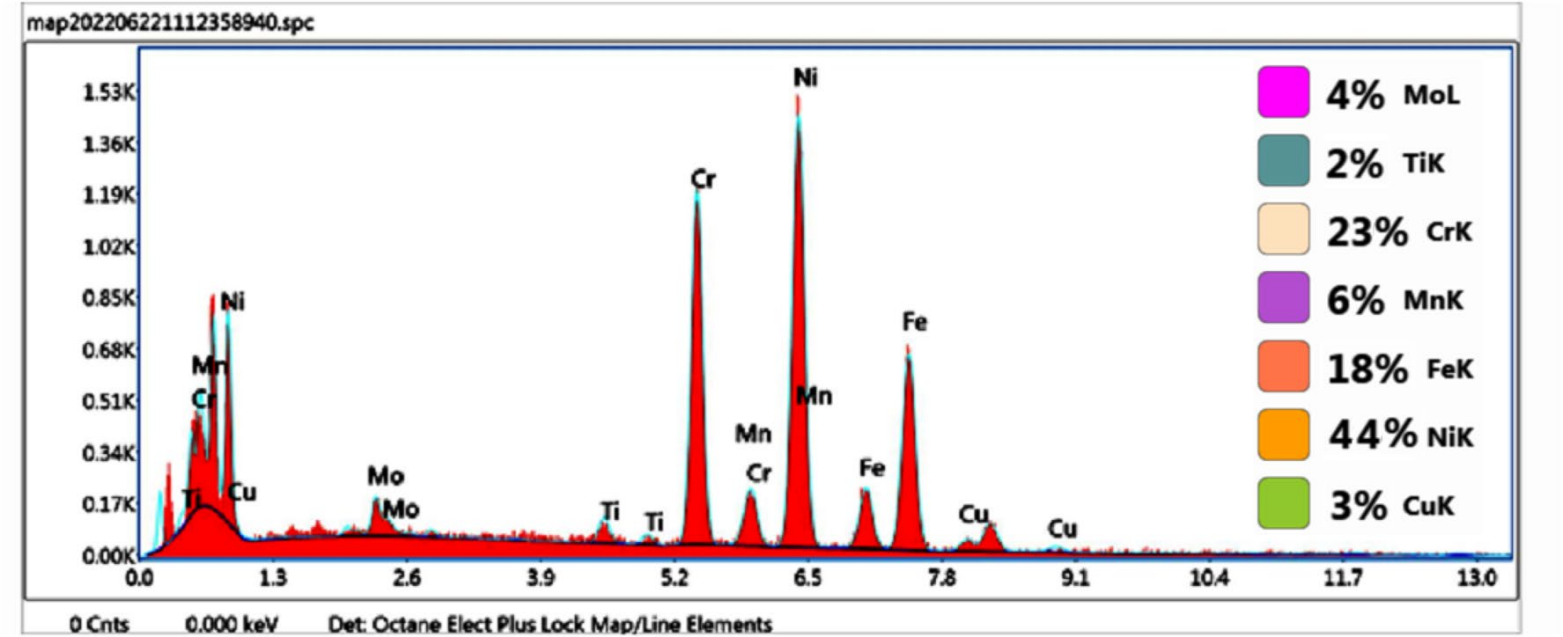
EDS Elemental Spectrum and Quantification of the Inconel 825 region.

The EDS analysis was carried out on the fractured surface of both the Inconel 825 and SS 316L specimens. The EDS maps (Fig. 11a–h) and spectra (Fig. 12) of the notched Inconel 825 region show an overall elemental composition of 44 wt% Ni, 23 wt% Cr, 18 wt% Fe, and other alloys. The mapping reveals that the fractured surface is dominated by elements like Ni, Cr, and Fe over the other elements.

**Figure 13:**
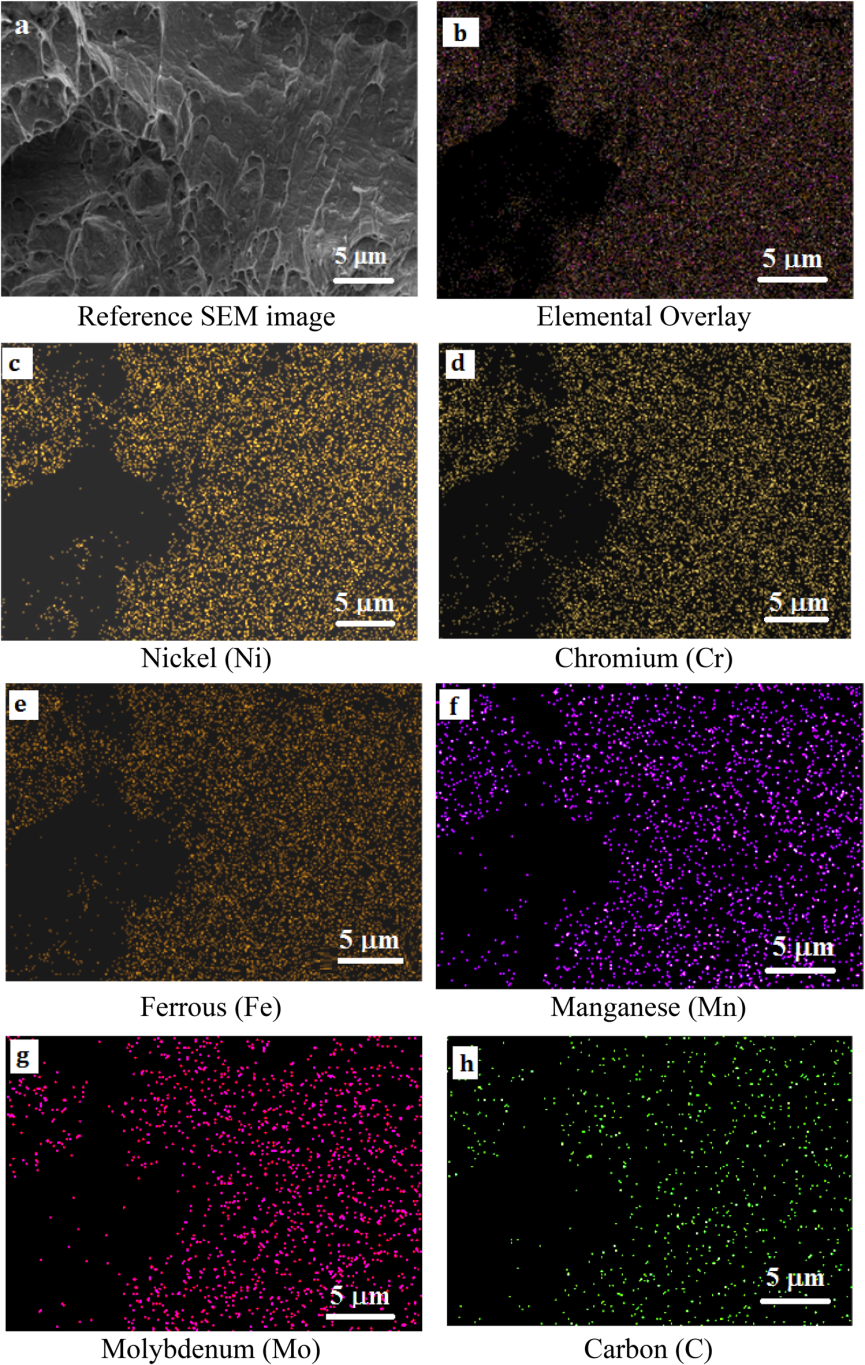
(**a**–**h**) EDS Elemental Mapping of the fractured SS316L region.

**Figure 14:**
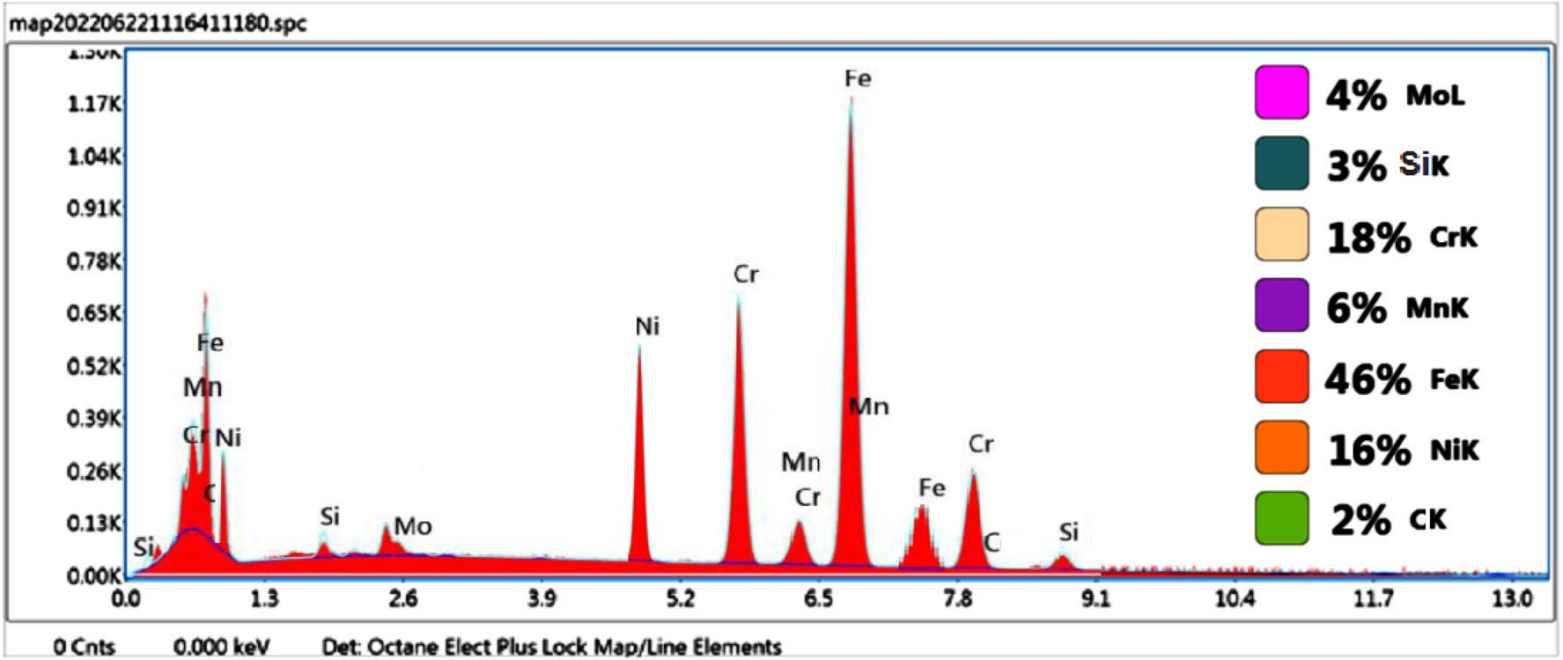
EDS Elemental Spectrum and Quantification of the SS316L region.

Similarly the EDS maps (Fig. 13a–h) and spectra (Fig. 14) of the notched SS316L region show an overall elemental composition of 16 wt% Ni, 18 wt% Cr, 46 wt% Fe, and other alloys. It was found that the composition of the fabricated wall is similar to the composition of the base metal, demonstrating the effective fabrication of the functionally graded wall with good properties.

In addition, the elemental maps confirm that there was no evidence of elemental segregation at the fractured surfaces of the notched Inconel 825 and SS316L samples and that the elements were dissolved evenly which confirms that the metals at the interface are strongly bonded.

## Conclusion

The CMT based WAAM process is used to construct functionally graded walls, and the metal transfer characteristics indicate the successful use of WAAM to produce structurally sound components. The fracture toughness of two functionally graded specimens with notches on the Inconel 825 and SS316L sides was assessed using the CTOD method and the SENB specimen geometry. The following conclusions are arrived:

1. The fabricated Inconel 825 specimen has both continuous and discontinuous cellular dendritic microstructures, whereas the SS316L specimen has austenite and 5% delta ferrite in its microstructure.
2. Both Inconel 825 and SS316L tensile fractograph revealed considerable plastic deformation, indicating a ductile mode of fracture.
3. The fracture toughness test results show that there is no considerable difference in the values of CTOD (0.853 mm for the Inconel 825 side and 0.873 mm for the SS316L side).
4. The values of fracture toughness differ by a significant margin from one another; Inconel 825 has a fracture toughness of 36 Mpa$\sqrt {\text{m}}$, while SS316L has a fracture toughness of 31.6 Mpa$\sqrt {\text{m}}$.
5. The fracture morphology of both the side-notched specimens indicates that they were fractured in the ductile mode with striations perpendicular to the direction of crack development.

Based on the findings of the research, the interface of the Inconel 825-SS316L wall has good fracture properties and can be used in harsh environments.

## Data Availability

All data generated or analysed during this study are included in this published article.
